# ELEVATE – evaluating Temozolomide and Nivolumab in patients with advanced unresectable previously treated oesophagogastric adenocarcinoma with MGMT methylation: study protocol for a single arm phase II trial

**DOI:** 10.1186/s12885-022-09891-9

**Published:** 2022-09-01

**Authors:** Elizabeth Smyth, Kelly Cozens, Daniel Griffiths, Kathryn L. Clark, Sean Ewings, Russell Petty, Tim Underwood, Rebecca C. Fitzgerald, James Tanner, Olivier Giger, Shubha Anand, Gareth Griffiths

**Affiliations:** 1 Cambridge University Hospitals National Health Service Foundation Trust, Hill’s Road, Cambridge, UK; 2 Southampton Clinical Trials Unit, University of Southampton and University Hospital Southampton NHS Foundation Trust, Southampton, UK; 3 University of Dundee, Dundee, UK; 4 School of Cancer Sciences, University of Southampton and University Hospital Southampton NHS Foundation Trust, Southampton, UK; 5 University of Cambridge, Cambridge, UK

**Keywords:** Oesophagogastric adenocarcinoma, Immunotherapy, MGMT methylated, Phase II

## Abstract

**Background:**

For patients with oesophagogastric adenocarcinoma, surgery is the only curative option and despite the use of multimodality therapy, which combines it with chemotherapy and/or radiotherapy, more than 50% of patients will relapse and die. Many UK patients present with advanced disease which is already inoperable or metastatic at diagnosis. For these patients, standard care chemotherapy only offers them survival of less than a year. Nivolumab, a checkpoint blockade inhibitor, has been found to work in some advanced cancers. It is proposed, for those where immunotherapy hasn’t worked, that these immunologically evasive tumours need to be sensitized to immunotherapy drugs to allow them to act.

**Methods:**

ELEVATE is a single arm phase II trial testing the overall response to nivolumab following temozolomide treatment in patients with advanced unresectable previously treated adenocarcinoma which is O^6^-methylguanine-DNA-methyltransferase (MGMT) methylated. 18 patients are being recruited from UK secondary care sites. To be eligible, participants must have been treated with at least 3 months of platinum and fluoropyrimidine chemotherapy. Participants will receive 50 mg/m^2^ temozolomide continuously for 3 months. If their disease progresses during the 3 months, they will stop temozolomide and start nivolumab at a dose of 240mg every 2 weeks. If there is no progression after 3 months the participant will continue taking temozolomide in combination with nivolumab. All treatment will stop once the participant progresses on nivolumab. The primary endpoint is the best overall response to nivolumab, using both Response Evaluation Criteria in Solid Tumours version 1.1 and immunotherapy modified Response Evaluation Criteria in Solid Tumours. Secondary endpoints include progression-free survival, overall survival, and quality of life.

**Discussion:**

ELEVATE will provide evidence for whether giving nivolumab after temozolomide in patients with previously treated advanced oesophagogastric adenocarcinoma is safe and biologically effective prior to future randomised trials.

**Trial registrations:**

EudraCT Number: 2020-004771-41(issued 01 October 2020); ISCRTN11398887(registered 14 July 2021).

**Supplementary Information:**

The online version contains supplementary material available at (10.1186/s12885-022-09891-9).

## Background

Gastric and oesophageal cancers are the 5th and 8th most commonly diagnosed cancers, respectively, and lead to more than 1.1 million deaths per year globally [1]. Although the incidence of distal gastric cancers has fallen sharply, the incidence of lower oesophageal and gastro-oesophageal junctional adenocarcinomas has risen rapidly in Western populations [2]. From 2014-2016 there were approximately 9,100 new oesophageal cancer cases in the UK every year and 7,900 oesophageal cancer deaths. In the same period, there were 6,700 new stomach cancer cases per year and around 4,500 stomach cancer deaths annually [3].

For patients with oesophagogastric adenocarcinoma (OGA), surgery offers the only chance of cure, however despite optimum multimodality therapy (surgery plus chemotherapy or chemoradiotherapy), more than 50% of patients relapse and die from metastatic disease [4–6]. Additionally, in the UK, most patients present with advanced, inoperable, or metastatic disease [3]. In advanced OGA (unresectable or metastatic cancer) treatment with the standard of care chemotherapy results in a median survival of less than one year in randomised trials, and although immunotherapy in combination with chemotherapy shows promise, the benefit of this approach appears to be limited to patients with high levels of PD-L1 expression [7–9]. Recommended standard of care chemotherapy in the first line setting usually includes a platinum and fluoropyrimidine, sometimes with the addition of a taxane or anthracycline; patients who have HER2 positive OGA are additionally offered trastuzumab [8–10]. In OGA, second line chemotherapy with a taxane or irinotecan results in an improvement in median overall survival of approximately six weeks in multiple international studies [11, 12]. The anti-VEGFR2 monoclonal antibody ramucirumab has monotherapy activity comparable to second line cytotoxic chemotherapy, and when added to paclitaxel, improves overall survival compared to paclitaxel alone, however ramucirumab is not funded in the UK for NHS patients [13, 14]. Therefore, novel approaches to improve survival for patients with advanced unresectable or metastatic OGA are desirable.

Immune checkpoint blockade therapy has revolutionised the treatment of multiple cancers including melanoma, lung, bladder and renal cancers [15–19]. Anti-PD-1 therapy has shown promise in the treatment of OGA patients. In the Asian ONO-4538-12 (ATTRACTION-2) trial, a phase III randomized study, 493 patients with chemo refractory unresectable advanced or recurrent oesophagogastric cancer were randomized 2:1 to either nivolumab 3mg/kg every two weeks or placebo [20]. Treatment with nivolumab improved median overall survival from 4.14 months to 5.32 months (HR 0.63; 95% CI (0.50-0.78), p<0.0001). Survival at 12 months was more than doubled for nivolumab treated patients (26.6% for nivolumab vs 10.9% for placebo). RECIST responses were observed in 12% of nivolumab patients, however tumour shrinkage was demonstrated in 40% of patients. A survival benefit for nivolumab treatment was observed for patients with and without PD-L1 expression (PD-L1 negative median OS 6.1 months vs. 4.2 months nivolumab vs placebo; PD-L1 positive median PS 5.2 months vs 3.8 months nivolumab vs. placebo). Thus, nivolumab is effective in chemo refractory gastric cancer independent of PD-L1 status. Similar results to those observed in Asian patients with single agent nivolumab in ATTRACTION-2 have been demonstrated in non-Asian patients in the CHECKMATE-032 study [21]. Pembrolizumab, a humanised immunoglobulin G4 monoclonal antibody targeting PD-1, has demonstrated similar results to nivolumab in OGA. In the non-randomised, international, KEYNOTE 059 study cohort 1,259 chemo refractory OGA patients (previously been treated with two or more lines of chemotherapy) were treated with pembrolizumab 200mg Q3W [22]. In KEYNOTE-059 cohort 1 RECIST responses were observed in 12% of all patients and 42% of patients had some evidence of tumour shrinkage, consistent with the results of nivolumab observed in the same setting [20, 21]. Following the results of ATTRACTION-2 and KEYNOTE-059 [22], nivolumab and pembrolizumab have been licensed in Asia and the USA for chemo refractory OGA (for PD-L1 positive patients only for pembrolizumab); however neither treatment is licensed in Europe for OGA patients.

Despite the early evidence of efficacy of anti-PD-1 therapy in chemo refractory OGA, immune checkpoint blockade inhibitors have not been successful in non-chemo refractory OGA disease settings in biomarker unselected populations. In the KEYNOTE 061 trial, pembrolizumab was compared to paclitaxel chemotherapy for second line OGA and failed to improve overall survival, either for PD-L1 positive (combine proportion score 1; primary endpoint) or PD-L1 negative cohorts [23]. However, a benefit of pembrolizumab compared to paclitaxel was observed for patients with microsatellite high tumours or who had tumour which expressed high levels of PD-L1 (Combined Proportion Score 10). In the first line KEYNOTE 062 trial, although pembrolizumab monotherapy was not inferior to chemotherapy in high PD-L1 (CPS 10 patients), in patients with low CPS scores neither pembrolizumab monotherapy, nor pembrolizumab plus chemotherapy were superior to chemotherapy alone [24]. Finally, in the CheckMate 649 trial, adding nivolumab to chemotherapy did extend overall survival, but only for patients with high PD-L1 expression (CPS 5) [9]. These findings emphasise that anti-PD-1 therapy is most effective in OGA patients who have been sensitised to immune checkpoint blockade therapy either through the presence of high mutation burden or other factors which lead to a robust immune infiltrate [25].

The enzyme O^6^-methylguanine-DNA-methyltransferase (MGMT) is responsible for removal of alkyl groups from the O^6^-position of guanine; when the MGMT promoter is methylated then protein expression is reduced and the capacity to repair O^6^-alkylguanine adducts is reduced, which can lead to increased sensitivity to alkylating agents. This is evidenced by modest radiological responses to single agent temozolomide (TMZ) or dacarbazine in MGMT methylated or protein negative tumours that do not characteristically respond when not biomarker selected (e.g. oesophageal or colorectal cancer (CRC) (9.4% ORR in oesophageal cancer) [26, 27]. Tumours which are MGMT methylated are sensitive to temozolomide.

Tumours which are MGMT deficient develop acquired mismatch repair deficiency as a resistance mechanism to TMZ treatment. In recent work, colorectal cell lines sensitive to TMZ (predominantly MGMT deficient) were treated with TMZ until resistance emerged and underwent molecular profiling. This revealed the frequent emergence of mismatch repair deficiency and a hypermutated state [28]. The same group went on to demonstrate that in a subset of CRC patients treated on clinical trials who initially benefited from TMZ and subsequently progressed, changes in mismatch repair (MMR) status were observed in the post-treatment biopsy in 40% of cases. The ELEVATE trial is the first to address this question in oesophagogastric adenocarcinoma patients.

TMZ is given on an intermittent or continuous basis, according to the indication, and regimens may have differential effects in evoking MMRd and on the immune microenvironment. For example, continuous dosing with TMZ can lead to T-reg depletion whereas standard dosing does not. T-reg depletion would be more favourable for a response to immune checkpoint blockade. Different TMZ dosing regimens also have different effects on lymphocytes, with consequent differences in antigen specific immune responses and outcomes both in the clinic and in animal models [29–35]. As continuous dosing is more likely to be better tolerated after first line chemotherapy due to low daily doses, this approach is favoured for the ELEVATE trial. TMZ will be dosed until progression, as the only practical clinical indication of TMZ resistance, required for the development of MMRd in experimental models [28].

## Methods/Design

The ELEVATE trial is a phase II trial evaluating whether Temozolomide and Nivolumab in patients with advanced unresectable previously treated oesophagogastric adenocarcinoma with MGMT methylation is safe and is biologically active.

## Objectives

The primary objective is to determine the activity of maintenance TMZ dosing followed by nivolumab treatment to evaluate the potential for a future randomised trial against a standard of care control arm. The rationale to continue TMZ for 3 months or until PD is to evaluate the emergence of Mismatch Repair Deficiency (MMRd) both with and without radiological PD, as clinically relevant MMRd may emerge before radiological progression. This will also reduce the number of patients who drop out due to symptomatic progressive disease.

Secondary objectives include determining the safety of TMZ and nivolumab, the effect of TMZ priming followed by nivolumab on disease control rates, progression free survival (according to RECIST v1.1 and iRECIST criteria), overall survival and safety and toxicity. The quality of life of trial participants will also be assessed using EORT-QLQ-C30, EORT-QLQ-OG25 and EQ-5D-5L questionnaires.

Exploratory objectives include identifying the proportion of patients treated with TMZ that develop MMRd, by comparing pre-TMZ and post-TMZ tumour biopsies. Understanding the relationship between MGMT protein expression, MGMT methylation and acquisition of MMRd, and the rate of increased acquisition of tumour mutation burden after TMZ and the relationship between Tumour Mutational Burden (TMB) acquisition and response to nivolumab will be explored. MGMT immunohistochemistry status after chemotherapy but before TMZ will be characterised. Another objective is to interrogate the response of the tumour microenvironment to immune checkpoint blockade. Tumour evolution of non-MMR genes will also be assessed under chemotherapy using archival, post-chemotherapy and post TMZ biopsies. ctDNA for tumour burden and clonal dynamics will be assessed comparing a baseline sample to post-TMZ and post nivolumab in relation to clinical and radiological response. We will correlate TMB in blood with TMB using Whole Genome Sequencing (WGS).

## Study design

**Fig. 1:**
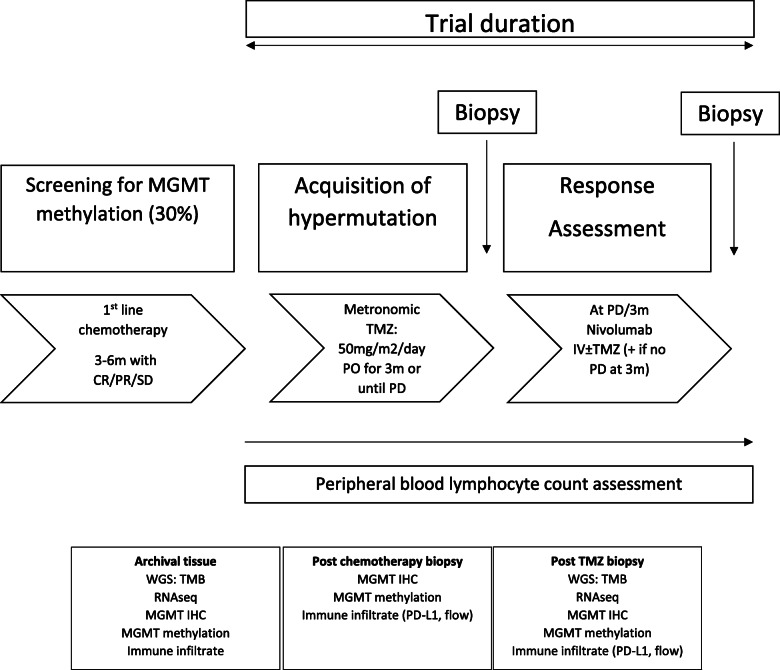
ELEVATE Trial schema

ELEVATE is a multicentre single arm Phase II trial in patients with advanced unresectable previously treated oesophagogastric adenocarcinoma with MGMT methylation (Fig. 1), using an A’Hern’s single stage design [36].

### Treatment

All participants will receive metronomic TMZ followed by nivolumab combination or monotherapy. Participants will receive TMZ 50 mg/m^2^/day for 3 months or until disease progression, determined by RECIST 1.1 criteria. TMZ dose will be rounded to the nearest 5 mg. TMZ is taken orally every day throughout the 28 day cycle. Capsules should be swallowed whole with a glass of water, at least one hour before or 2 hours after food. Missed doses of more than 12 hours late should not be taken. Participants treated with TMZ should receive 960 mg co-trimoxazole three times per week as prophylaxis against opportunistic infections. Anti-emetics can be given as per local practice. Participants should maintain a normal diet unless modifications are required to manage an AE such as diarrhoea, nausea or vomiting.

After 3 months of TMZ or following progression on TMZ (or other withdrawal from TMZ due to, for example, toxicity), whichever comes first, all participants will then receive nivolumab at 240 mg as a 30 minute infusion every 2 weeks +/- TMZ (where TMZ will only continue if the participant has not progressed or withdrawn from TMZ treatment). Treatment with nivolumab will commence within 3 weeks of TMZ progression or month 3 of TMZ treatment. Nivolumab +/- TMZ will continue until definitive disease progression (determined by iRECIST), a maximum of 24 months, death, or withdrawal from treatment or study. The Schedule of Events (Table 1) details the trial treatment schedule.

**Table 1:** Schedule of observations and procedures (SPIRIT figure for ELEVATE – Additional file 4)

Visit:	Pre-Screen	Screening (within 28 days of C1)	Baseline (D1 C1 TMZ – 28 day cycle)	Day 1 of each TMZ cycle	Progression or cessation of TMZ or at month 3/day 84 (+/- 7 days)	Day 1 of each Nivo (+/- TMZ) cycle	Progression/End of treatment
Informed consent - Tumour analysis for MGMT methylation	X						
Archival tumour tissue to central laboratory	X						
Informed Consent		X					
Eligibility Evaluation		X					
Medical History		X					
Mismatch repair proficient testing (MSI-normal or MMR intact)		X					
Physical Exam^a^		X	X	X	X	X	X
Vital signs^b^		X	X	X	X	X	X
ECOG performance Status		X	X	X	X	X	X
Phone call to check compliance				X		X	
Echo/MUGA^c^		X					
Pregnancy test in WOCBP		X	X	X		X	
CT Scan^d^		X		X^e^	X^f^	X^g^	
Serum Chemistry (U&E^h^, LFTS^i^,CalciumCA19-9)		X	X	X	X	X	X
Endocrine tests: Morning cortisol, ACTH, TSH (Free T3 and Free T4 in case of abnormal TSH)		X				X^j^	
Full blood count		X	X^k^	X^k^	X	X^k^	
Serology – HBV or HCV		X					
ECG^l^		X					
Adverse Events		X	X	X	X	X	X
Concomitant Medication		X^m^	X	X	X	X	X
Patient registered with NHS Digital			X				
Quality of Life Questionnaires^n^			X	X	X	X	X
Details of next treatment							X
TRANSLATIONAL SAMPLES							
Tumour Biopsy (endoscopy)					X		X^o^
Translational blood (ctDNA, Plasma)		X	X	X^p^	X	X^q^	X

^a^Height and weight. Height only required at screening

^b^Temperature, blood pressure, heart rate, respiratory rate, oxygen saturation

^c^If clinically indicated

^d^Assessments performed following RECIST 1.1/iRECIST guidelines as appropriate)

^e^CT scans are required at week 6 and week 12 of TMZ treatment. Assessment of scan should be completed following RECIST 1.1 guidelines

^f^CT scan not required if cessation of TMZ occurs within 4 weeks of the week 6 scan

^g^CT scan at week 6 and week 12 of Nivolumab treatment and then every 12 weeks thereafter until CT scan demonstrates unconfirmed progression, following iRECIST guidelines, then a confirmatory CT scan should be performed 4-6 weeks later

^h^Urea and electrolytes

^i^AST and/or ALT, ALP, bilirubin

^j^Every 6 weeks on nivolumab (TSH, cortisol)

^k^FBC weekly whilst on TMZ

^l^Within 7 days prior to treatment start date

^m^To include all those being taken from 30 days prior to starting trial treatment and during treatment and up to 100 days after last study dose

^n^EORTC QLQ-C30, EQ-5D-5L & QLQ-OG25

^o^Optional endoscopy

^p^At 4-6 weeks from D1 C1 TMZ

^q^At 4-6 weeks from D1 C1 Nivolumab

There will be no dose escalations or reductions of nivolumab allowed. Participants may be dosed no less than 12 days from the previous dose. The infusion will be administered through a sterile, non-pyrogenic, low protein binding in-line filter with a pore size of 0.2-1.2 *μ*m. It will not be administered as an intravenous push or bolus injection. The total dose may be infused directly as a 10 mg/mL solution or can be diluted to as low as 1 mg/mL with sodium chloride 9 mg/mL (0.9%) solution for injection or glucose 50 mg/mL (5%) glucose solution for injection. The infusion will be administered over 30 minutes. After administration the line will be flushed with sodium chloride 9 mg/mL (0.9%) solution for injection or 50 mg/mL (5%) glucose solution for injection.

During continuous dose TMZ, the participants’ FBC should be checked weekly. TMZ can continue, providing the following conditions are met:

- Absolute Neutrophil Count (ANC) 1.5 x 10^9^/l
- Thrombocyte count 100 x 10^9^/l
- Common toxicity criteria (CTC) non-haematological toxicity Grade 1 (except for alopecia, nausea and vomiting).

Table 2 details the criteria for modifying the continuous dosing of temozolomide.

**Table 2:** Dose modifications for continuous dosing temozolomide

Toxicity	Withhold TMZ (until counts return to above criteria)	TMZ discontinuation
ANC	0.5 and < 1.5 x 10^9^/l	< 0.5 x 10^9^/l
Platelets	10 and < 100 x 10^9^/l	< 10 x 10^9^/l
CTCAE toxicity (version 5)	Grade 2	Grade 3-4

While dose modifications are not permitted on this trial, there are a set of criteria that should be assessed when considering delaying, discontinuing or restarting nivolumab treatment in relation to adverse events and infusion related events (Additional file 1 explains these criteria in more detail).

Participants on IV steroids may be switched to an equivalent dose of oral corticosteroid (e.g. prednisolone) at start of tapering or earlier, once sustained clinical improvement is observed. Lower bioavailability of oral corticosteroids should be considered when switching to the equivalent dose of oral corticosteroids.

Prophylactic antibiotics should be considered in the setting of ongoing immunosuppression.

### Setting

ELEVATE will be run in 6-8 secondary care hospitals in the UK with the aim of recruiting up to a total of 18 evaluable patients.

### Sample size and recruitment

The sample size calculation was performed using Sample Size Tables for Clinical Studies. This phase II trial will be based on A’Hern’s single stage design [36]. The primary endpoint of objective response measured in ELEVATE is to nivolumab (following progression, or 3 months without progression, on TMZ), not to TMZ monotherapy, i.e., the baseline measurement for indicating response/progression is taken from the point of initiation of nivolumab.

Based on 10% alpha and 80% power, and assuming a response rate of <10% would not be sufficient activity to warrant further investigation in a future phase III, and a >30% response rate would warrant further investigation in a future phase II, the required sample size is 18. If 4 or more responses are observed in 18 patients then the treatment will be considered sufficiently active to justify its inclusion in a future phase III. The lower limit of 10% ORR was chosen based on this being expected when using nivolumab in unselected populations.

In order to identify 18 MGMT low/methylated patients and based on an estimated minimum 20% showing MGMT loss then, 90 patients will be required to be screened. We anticipate needing to screen approximately 120 patients to account for screen failures and a very small number of deaths in recruited participants prior to starting nivolumab.

## Ethical and regulatory aspects

The study received ethical approval from East of England – Cambridge South Research Ethics Committee on 15-Mar-2021 (ref: 21/EE/0017) and has Health Research Authority (IRAS 282284) and UK Medicines and Health Care Product Regulatory Agency (MHRA) approvals. Southampton Clinical Trials Unit (SCTU), a Cancer Research UK core funded and UK Clinical Research Collaboration registered Clinical Trials Unit (CTU), is coordinating the trial. A list of recruiting sites can be obtained from the SCTU. The University of Southampton is the sponsor for the trial and is responsible for all legal requirements of conducting a non-commercial clinical trial of an investigational product. The drug for this trial is provided by Bristol Myers-Squib (BMS) who are responsible for the Good Manufacturing Practice quality drug and who is also providing research funding.

The ELEVATE Trial Management Group (TMG) is responsible for overseeing the progress of the trial, including both the clinical and practical aspects. The Chair of the TMG will be the Chief Investigator of the trial. The TMG includes representatives with expertise in oncology, radiology, translational science and medical statistics; as well as being supported by a Patient and Public Involvement contributor and CTU staff involved in the day-to-day running of the trial. An independent Trial Steering Committee has also been established, and an independent DMEC comprising three clinicians and a statistician experienced in this research area (but not directly involved in this trial apart from DMEC membership) has been set up. The aim of the independent DMEC is to safeguard the interests of trial participants, monitor the main outcome measures including safety and efficacy, and monitor the overall conduct of the trial. Charters for these groups are available via elevate@soton.ac.uk .

The SCTU has undertaken a risk assessment for the ELEVATE trial, which includes the requirements for monitoring (both central and site). The SCTU undertakes several internal audits of its own systems and processes annually and has routine audits from both its sponsor and the independent MHRA every 2-3 years.

## Study participants

The ELEVATE trial is currently recruiting patients with advanced unresectable previously treated oesophagogastric adenocarcinoma with MGMT methylation for which the use of TMZ followed by nivolumab is clinically appropriate treatment in the view of the local principal investigator. The previous treatment must include at least 3 months of platinum and fluoropyrimidine based chemotherapy for advanced disease (see Table 3 for full eligibility criteria). No other investigational medicinal products should be received whilst on study. Immunosuppressive agents, immunosuppressive doses of systemic corticosteroids, concurrent anti-neoplastic therapy and live vaccinations are prohibited during the study. Caution must be used with ototoxic or nephrotoxic concomitant drugs and herbal medication should be stopped prior to study enrolment.

**Table 3:** Eligibility Criteria for the ELEVATE Trial

Inclusion Criteria
1. Participants 18 years of age
2. Pathologically confirmed advanced unresectable or metastatic OGA
3. MGMT methylation on archival tissue
4. Mismatch repair proficient (MSI-normal or MMR intact)
5. Previously treated with at least 3 months of platinum and fluoropyrimidine based chemotherapy for advanced disease, without evidence of disease progression
6. Measurable disease per RECIST 1.1 guidelines
7. ECOG Performance Status of 0 or 1
8. Can swallow TMZ capsules
9. Adequate organ function assessed within 7 days before randomisation:
∙ White blood cell count (WBC) > 1.5 X 10^9^/L
∙ Absolute neutrophil count (ANC) > 1.5 X 10^9^/L
∙ Platelets 100 X 10^9^/L
∙ Haemoglobin 90 g/l
∙ Measured/calculated creatinine clearance 60 mL/min (according to Cockroft-Gault formula)
∙ Total bilirubin within normal limits (if patient has documented Gilbert’s disease 1.5 X ULN or direct bilirubin 1.5 X ULN)
∙ Aspartate transaminase (AST) and/or alanine transaminase (ALT) 1.5 X ULN
10. All toxicities (except alopecia, and grade 2 fatigue, neuropathy and lack of appetite/nausea) attributed to prior anti-cancer therapy must have resolved to grade 1 (NCI CTCAE version 5.0) or baseline before administration of study drug
11. Women of childbearing potential (WOCBP) may be included following a confirmed menstrual period and must have a negative serum or urine pregnancy test (minimum sensitivity 25 IU/L or equivalent units of human chorionic gonadotropin (HCG)). The pregnancy test must be within 24 hours prior to starting treatment
12. WOCBP should use one highly effective and one effective method of birth control during the study treatment period and for at least 5 months after the last dose of study treatment
13. Female subjects who are breastfeeding should discontinue nursing prior to the first dose of study treatment and until 5 months after the last dose of study treatment
14. Men who are sexually active with a WOCBP must adhere to contraception during and for a period of 7 months after the last dose of study treatment
15. Absence of any psychological, familial, sociological or geographical conditions potentially hampering compliance with the study protocol and follow-up schedule; those conditions should be discussed with the patient before registration in the trial
16. Written informed consent
Exclusion Criteria
1. Previous treatment with TMZ
2. Prior treatment with an anti-PD-1, anti-PD-L1, anti-PD-L2, anti-CD137, or anti-CTLA-4 antibody, or any other antibody or drug specifically targeting T-cell co-stimulation or immune checkpoint pathways
3. Active central nervous system metastases
4. Candidate for curative surgery
5. Previous malignancies are excluded unless a complete remission was achieved at least 5 years prior to study entry. Adequately treated cervical carcinoma in situ, and localised non-melanoma skin cancer are not exclusion criteria, regardless of timepoint diagnosis
6. Active, known, or suspected infectious or autoimmune disease (except for patients with type 1 diabetes mellitus, residual hypothyroidism due to autoimmune thyroiditis only requiring hormone replacement, skin disorders (such as vitiligo, psoriasis, or alopecia) not requiring systemic treatment are permitted to enrol)
7. Conditions requiring systemic treatment with either corticosteroids (10 mg daily prednisolone or equivalent) or other immunosuppressive medications within 14 days of study drug administration
8. Interstitial lung disease
9. > Grade 1 peripheral neuropathy
10. Positive test result for HBV or HCV indicating acute or chronic infections
11. Known history of testing positive for HIV or known AIDS
12. Known hypersensitivity to the components or excipients of co-trimoxazole, temozolomide or nivolumab
13. Known hypersensitivity to dacarbazine (DTIC)
14. Clinically significant abnormal 12-lead ECG. If clinically indicated, cardiac function assessment using either echocardiography or MUGA Scan, if clinically significant the patient is ineligible
15. In the past 6 months serious cardiac illness or medical condition including but not confined to:
∙ History of documented CHF
∙ High-risk uncontrolled arrhythmias
∙ Angina pectoris requiring antianginal medication
∙ Clinically significant valvular heart disease
∙ Evidence of transmural infarction
∙ Poorly controlled hypertension (e.g. systolic >180mm Hg or diastolic > 100mm Hg)
16. Patients with severe liver parenchymal damage
17. Patients with severe renal insufficiency where repeated measurements of the plasma concentration cannot be performed
18. Patients with a history of drug-induced immune thrombocytopenia with use of trimethoprim and/or sulfonamides
19. Patients with acute porphyria
20. Patients with severe myelosuppression

### Withdrawal criteria

Participants are free to withdraw consent from the study at any time without providing a reason. A participant could also withdraw from receiving study treatment but may not wish to withdraw from the trial. In this instance, the participant will be encouraged to attend follow-up visits in accordance with the trial schedule. Should any participant become pregnant during the trial, study treatment will be discontinued.

After the participant has entered the trial, the clinician remains free to give alternative treatment to that specified in the protocol at any stage if they feel that it is in the participant’s best interest, but the reasons for doing so should be recorded. In these cases, participants will be withdrawn from protocol treatment but remain within the trial for the purposes of follow-up and data analysis. All participants are free to withdraw at any time from the protocol treatment without giving reasons and without prejudicing further treatment.

## Study procedure

### Tumour analysis consent

There will be a two-stage consent process for participation in the ELEVATE trial. Stage 1 will ask the patient to consent for their archival tumour or resection sample, collected at diagnosis, to be analysed. The sample will be sent to the central laboratory. Should the analysis show MGMT methylation, the patient will be asked if they wish to participate in the main trial. Before consent is obtained, the patient will be given a full explanation of this stage of the trial and offered a Patient Information Sheet (PIS). Patients can consent to stage 1 by phone (sending the Stage 1 Informed Consent Form (Additional file 2) by post to the research office) or in person. To be eligible to consent to stage 1, patients must have:

- Any advanced OG cancer, planned to be treated or being treated, with at least 3 months of platinum and fluoropyridine based chemotherapy
- Archival tumour tissue available to be sent to the central laboratory for analysis

### Main trial consent

If MGMT methylation is confirmed, the patient will be asked to consent to the main trial. Consent to enter the main trial will be sought from each participant only after a full explanation has been given, a PIS offered and time allowed for consideration. Signed participant consent will be obtained, using the trials Informed Consent Form (Additional file 3), within a hospital setting. The process will be documented in the patient’s medical records. Only site staff named on the Delegation Log and authorised to do so may obtain consent. Patients may refuse to participate without giving reasons and this will not prejudice their future treatment.

The trial’s Informed Consent Form detail the consent provisions for collection and use of participant data and biological specimens in future research (Additional files 2 and 3).

### Screening

Following informed consent for the main trial, assessments including a physical examination, full blood count, serum biochemistry, including renal, liver and bone profiles, HBV, HCV and HIV serology, and endocrine tests (morning cortisol, ACTH, TSH (Free T3 and Free T4 in case of abnormal TSH)) are completed within 28 days prior to TMZ treatment commencing, with a CT scan being undertaken to assess the disease according to RECIST v1.1. Concomitant medication and medical history will be recorded. In addition, women of childbearing potential (WOCBP) will undertake a pregnancy test. An ECG will be carried out, as will an Echo/MUGA, if clinically indicated. Bloods for translational research will also be collected during screening.

### Treatment and follow-up visits

Participants attend hospital appointments for treatment cycles with assessments similar to those performed during screening, plus reviews of adverse events and treatment adherence. The investigator and/or study personnel will phone participants to check for adherence and toxicities to TMZ, and participants will also have weekly FBC. The time schedule of enrolment, interventions, assessments, and visits for participants are fully detailed in the schedule of events (Table 1). Participants completing the trial, defined as progression on nivolumab (following iRECIST criteria), unacceptable toxicity, withdrawal of consent, or the maximum treatment duration of 24 months on nivolumab, will be managed by standard clinical care from their usual treating clinician. Following confirmed progression patients will be followed up remotely every 4 weeks (via a patient notes check or phone call if required) for details of next treatment and survival until the last participant has completed the study.

Serious adverse event reporting occurs in real-time to the SCTU safety desk throughout the study. Serious adverse events are assessed to determine whether they are related to drug treatment and unexpected or not, and subsequently reported to both Bristol Myers Squibb and the UK regulatory bodies.

### Translational research

Tumour samples will be examined for biomarkers (proteins, DNA and RNA) which might be associated with sensitivity to TMZ and nivolumab. Plasma samples will be examined for ctDNA.

### Contraception

Contraception is mandated from trial entry until at least 5 months (females of childbearing potential) or 7 months (male) following the last dose of study treatment.

Female participants who are of child bearing potential must have a negative serum or urine pregnancy test 24 hours prior to the start of treatment and agree to use two effective forms of contraception, one of which should be highly effective. These must be used from the first administration of study treatment, throughout the trial and for 5 months after last dose of study treatment.

A woman is considered of childbearing potential, i.e. fertile, following menarche and until becoming post-menopausal unless permanently sterile. Permanent sterilisation methods include hysterectomy, bilateral salpingectomy and bilateral oophorectomy. A postmenopausal state is defined as no menses for 12 months without an alternative medical cause. A high follicle stimulating hormone (FSH) level in the postmenopausal range may be used to confirm a postmenopausal state in women not using hormonal replacement therapy. However, in the absence of 12 months of amenorrhea, a single FSH measurement is insufficient.

Female patients must refrain from egg donation from the first administration of the study treatment, throughout the trial and for 5 months after the last dose of study treatment.

Male participants with female partners of child-bearing potential must agree to take measures not to father children by using one form of effective contraception (as above) to be effective from the first administration of all study drugs, throughout the trial and for 7 months after last dose of study treatment. Male participants must also refrain from donating sperm during this period.

Men with pregnant or lactating partners will be advised to use barrier method contraception (for example, condom plus spermicidal gel) to prevent exposure to the foetus or neonate.

## Data collection and management

### Plans for assessment and collection of outcomes

Hospital research staff will enter participant data into the study electronic case report forms (eCRFs) via a remote data collection tool (Medidata Rave). Only trained personnel with specific roles in the study will be granted access to the eCRFs. SCTU trial staff will regularly check the data for missing or anomalous values. Data queries will either be automatically generated within the eCRF, or manually raised with site by the SCTU team. Site staff will respond to explain or resolve the discrepancies.

The PIS and Informed Consent Form will outline the participant data to be collected and how it will be managed or might be shared, including handling of all Patient Identifiable Data (PID) and sensitive PID adhering to relevant data protection law.

### Data management

Participant data will be entered remotely at site and retained in accordance with the current Data Protection Regulations. The PI is responsible for ensuring the accuracy, completeness, and timeliness of the data entered.

The participant data is pseudo anonymised by assigning each participant a participant identifier code which is used to identify the participant during the trial and for any participant-specific correspondence between the SCTU and site. The site retains a participant identification code list, which is only available to site staff.

Only the investigator and personnel authorised by them should enter or change data in the eCRFs. When requested, laboratory data must be transcribed, with all investigator observations entered into the eCRF. The original laboratory reports must be retained by the Investigator for future reference.

Data queries will either be automatically generated within the eCRF, or manually raised by the SCTU, if required. All alterations made to the eCRF will be visible via an audit trail which provides the identity of the person who made the change, plus the date and time.

At the end of the trial, after all queries have been resolved and the database frozen, the PI will confirm the data integrity by electronically signing all the eCRFs. The eCRFs will be archived according to SCTU policy and a PDF copy including all clinical and Meta data returned to the Investigator for each participant.

## Oversight and monitoring

The trial may be subject to inspection and audit by University of Southampton (under their remit as Sponsor), SCTU (as the Sponsor’s delegate) and other regulatory bodies to ensure adherence to the principles of GCP, Research Governance Framework for Health and Social Care, applicable contracts/agreements and national regulations.

Upon receipt of a request from SCTU, the PI will allow the SCTU direct access to relevant source documentation for verification of data entered onto the eCRF (taking into account data protection regulations). Access should also be given to trial staff and departments (e.g. pharmacy). Where on-site monitoring is not permitted due to the COVID-19 pandemic, alternative strategies such as remote monitoring will be deployed. These will be fully described in the trial monitoring plan.

The participants’ medical records and other relevant data may also be reviewed by appropriate qualified personnel independent from the SCTU appointed to audit the trial, including representatives of the Competent Authority. Details will remain confidential and participants’ names will not be recorded outside the trial site without informed consent.

## Statistical analysis

For the primary and secondary objectives, patients who have consented and received at least one dose of nivolumab will be analysed. However, for the exploratory objectives those that have just received TMZ, and not commenced onto nivolumab, will also be analysed.

Response rates (including the primary endpoint) and other categorical data will be presented as percentages with 95% Clopper-Pearson confidence Intervals [37], with continuous data as means and standard deviations or medians and quartiles as appropriate. The primary endpoint will also be presented with a one-sided 90% lower confidence interval limit (to align with the sample size calculation). A secondary analysis will be conducted for the primary endpoint using only those who receive at least two weeks of TMZ (to acknowledge that this subgroup should have been exposed sufficiently for the proposed TMZ mechanisms to have had an impact). Time to event data will be presented in a Kaplan-Meier curve with median survival rates being presented. Waterfall plots will be produced to show changes in tumour size from baseline to treatment assessment time points.

A full and detailed statistical analysis plan will be developed prior to the final analysis of the trial.

All analyses will be carried out using STATA v16 or higher and/or SAS v9.4 or higher.

There are no plans for an interim analysis to be conducted during the ELEVATE trial.

## Adverse event reporting and harms

Data on AEs will be collected at treatment and follow-up visits. SAEs will be reported up to 100 days after the last administration of the trial drug to the SCTU safety desk. The trial also has a UK regulatory compliant real-time SAE reporting process to identify serious AEs and suspected unexpected SAEs that could suspend or stop the trial if warranted.

## End of the trial

The end of trial is defined as the last data collection from the last participant in the trial, required to answer the research question. This will be when the last participant concludes their nivolumab treatment, thus completing the trial. Participants will stop nivolumab treatment after progression (per iRECIST criteria), unacceptable toxicity, withdrawal of consent, or the maximum treatment duration of 24 months on nivolumab.

## Discussion

ELEVATE aims to determine whether giving nivolumab after temozolomide reduces tumour size as well as investigating the potential side effects in patients with previously treated advanced oesophagogastric adenocarcinoma. If ELEVATE demonstrates that this combination is safe and reduces tumour size it may pave the way for future phase III trials in patients with advanced unresectable previously treated oesophagogastric adenocarcinoma.

## First use of drugs in combination

This is the first time that this combination has been used in this patient population. Both drugs however have been used both on their own or in other combinations therefore there is a lot known about the safety profiles of each. The patients will be closely monitored during treatment for adverse events caused by the either drug. While on TMZ treatment participants will have their haematology bloods monitored weekly to check for adverse events. Trial safety data will be closely reviewed by the DMEC.

## Risk and benefits

Patients eligible for participation in ELEVATE will already have been treated with standard first line chemotherapy for OGA. There is no current recommendation for maintenance treatment following first line chemotherapy for OGA patients and thus these patients would not be recommended to have any active treatment until progression onto second line chemotherapy. Therefore, patients are not excluded from any standard treatment by participating in the trial. Participants in ELEVATE will be treated with temozolomide, which has demonstrated early evidence of activity in MGMT methylated patients, and subsequently will be treated with nivolumab, which has already shown efficacy and benefit for chemo refractory OGA patients. Patients treated with temozolomide, nivolumab or the combination may experience toxicity due to these treatments. However, toxicity and patient safety will be closely monitored, extensive toxicity management guidelines are provided (detailed in Additional file 1) to mitigate this issue. The non-clinical safety profile and emerging clinical profile, from studies using these drugs, have not identified risks that would preclude investigation in this setting. Finally, although there can be no certainty of clinical benefit to patients, it is hoped that the combination of temozolomide and nivolumab will improve outcomes for this group of patients with historically poor survival. Considering all these factors, the risk-benefit assessment for patient participation in the ELEVATE trial is assessed to be favourable as the treatment has the potential to offer a new and safe treatment strategy for advanced OGA patients.

## COVID-19

Chemotherapy and immunotherapy have not been shown to increase the risk of severe illness due to COVID-19 [38], however this combined regimen has not been specifically tested. The COVID risk associated with extra visits to the hospital for trial purposes will be discussed with the patient, however, the potential benefit of this intervention in the advanced disease setting could outweigh such risks. During a study visit participants will be instructed to adhere to local trust and national COVID-19 guidelines. There is no contra-indication to COVID vaccination before or during the study, although vaccination will not be mandated for study entry.

## Biological samples to be taken specifically for this study

Multiple blood samples will be taken in this study. Participants may also be subjected to up to two tumour biopsies. These study procedures have been designed to be as limited as possible to gain the necessary information and to be conducted in the least distressing way by experienced health care professionals.

## Ionising radiation

Participants will have CT scans at regular intervals throughout the trial (see table 1) to monitor the extent of the disease following RECIST v1.1 and iRECIST criteria. Apart from two, these scans are standard of care in OGA. Patients will be made fully aware of this and the risks of these scans prior to giving consent to their participation in the trial.

In addition, an Echocardiogram or MUGA may be performed if clinically indicated to establish whether patients are eligible for the trial.

## Trial status

The trial opened to recruitment on 23-Sep-2021. 18 patients will be recruited over an 18 month recruitment period at 6-8 sites in the UK.

This clinical trial was entered into EudraCT on 1^st^ October 2020 (EudraCT Number: 2020-004771-41) and registered on ISRCTN and ClinicalTrials.gov on 14^th^ and 30^th^ July 2021, respectively (ISRCTN11398887; ClinicalTrials.gov reference: NCT04984733). The current protocol is version 3 dated 13-May-2021. REC/MHRA approved protocol amendments will be communicated to sites via email and updated trial documentation provided centrally via the trial website. Trial registries will be amended where relevant with explanations for these changes.

## Supplementary Information

**Additional file 1** (Nivolumab Treatment Management) PDF Format. Criteria that should be assessed when considering delaying, discontinuing or restarting nivolumab treatment in relation to adverse events and infusion related events.

**Additional file 2** (Informed Consent Form for Tumour Analysis) PDF Format. Copy of the consent form given to participants for tumour analysis.

**Additional file 3** (Informed Consent Form for the Main T rial) PDF Format. Copy of the consent form given to participants for the main trial.

**Additional file 4** (The SPIRIT Checklist) PDF Format. Standard Protocol Items: Recommendations for Interventional Trials (SPIRIT): a checklist for a set of scientific, ethical and administrative elements recommended to be listed in a protocol [39].

## Data Availability

Pseudonymised individual participant data within the clinical trial dataset will be available for sharing via controlled access by authorised SCTU staff (as delegated to SCTU by the trial sponsor). Data access can be requested via a SCTU Data Release application form ; detailing the specific requirements and the proposed research, statistical analysis, publication plan and evidence of research group qualifications. Please email the completed form to the SCTU Data Release Committee Coordinator at ctu@soton.ac.uk. Data access requests are reviewed against specific eligibility criteria by the SCTU data custodian and key members of the trial team, including a statistician and chief investigator or by an external Independent Review Panel. Decisions about requests are made promptly and usually no more than three months after receipt of request. Responses to all data requests, with a clear rationale for any refusals, will be sent promptly to the data requester.

## Abbreviations

ANC: Absolute Neutrophil Count
AE: Adverse Event
BMS: Bristol Myers Squibb
CHF: Congestive Heart Failure
CI: Confidence Interval
CRC: Colorectal Cancer
CRF: Case Report Form
CTCAE: Common Terminology Criteria for Adverse Events
ctDNA: circulating tumour DNA
DMEC: Data Monitoring and Ethics Committee
DMP: Data Management Plan
DTIC: dacarbazine
ECOG: Eastern Cooperative Oncology Group
FSH: follicle stimulating hormone
GCP: Good Clinical Practice
HER2: Human Epidermal Growth Factor Receptor 2
IHC: Immunohistochemistry
IMP: Investigational Medicinal Product
iRECIST: immunotherapy modified Response Evaluation Criteria in Solid Tumours
LFTs: Liver Function Tests
MGMT: O^6^-methylguanine-DNA-methyltransferase
MHRA: Medicines and Healthcare products Regulatory Agency
MTIC: 5-(3-methyltriazen-1-yl) imidazole-4-carboxamide
MMR: Mismatch Repair
MMRd: Mismatch Repair Deficiency
MUGA: Multigated Acquisition
NCI: National Cancer Institute
OGA: Oesophago-Gastric Adenocarcinoma
ORR: Overall Response Rate
OS: Overall Survival
PFS: Progression Free Survival
PID: Patient Identifiable Data
REC: Research Ethics Committee
RECIST: Response Evaluation Criteria in Solid Tumours
SAE: Serious Adverse Event
SCTU: Southampton Clinical Trials Unit
U&E: Urea and Electrolytes
TMB: Tumour Mutational Burden
TMG: Trial Management Group
TMZ: Temozolomide
TSC: Trial Steering Committee
VEGFR: Vascular Endothelial Growth Factor Receptor
WBC: White Bloods Cells
WGS: Whole Genome Sequencing
WOCBP: Women of child bearing potential
