# Supplementary material for: ELEVATE – evaluating Temozolomide and Nivolumab in patients with advanced unresectable previously treated oesophagogastric adenocarcinoma with MGMT methylation: study protocol for a single arm phase II trial
Source: BMC Cancer. 2022 Sep 1;22:946. doi: 10.1186/s12885-022-09891-9 (PMC9434527; doi:10.1186/s12885-022-09891-9)
Supplement: Supplementary file 1 — Additional file 1 (Nivolumab Treatment Management) PDF Format. Criteria that should be assessed when considering delaying, discontinuing or restarting nivolumab treatment in relation to adverse events and infusion related events. [file 12885_2022_9891_MOESM1_ESM.pdf]

# Nivolumab Treatment Management

**Dose Delay/Discontinuation Criteria for Nivolumab**

| Immune-related adverse reaction | Severity | Treatment modification |
|---|---|---|
| Immune related pneumonitis | Grade 2 pneumonitis | Withhold dose(s) until symptoms resolve, radiographic abnormalities improve, and management with corticosteroids is complete. |
| | Grade 3 or 4 pneumonitis | Permanently discontinue treatment. |
| Immune-related colitis | Grade 2 diarrhoea or colitis | Withhold dose(s) until symptoms resolve and management with corticosteroids, if needed, is complete. |
| | Grade 3 diarrhoea or colitis<br>Nivolumab monotherapy | Withhold dose(s) until symptoms resolve and management with corticosteroids is complete. |
| | Grade 4 diarrhoea or colitis | Permanently discontinue treatment. |
| Immune-related hepatitis | Grade 2 elevation in aspartate aminotransferase (AST), alanine aminotransferase (ALT), or total bilirubin | Withhold dose(s) until laboratory values return to baseline and management with corticosteroids, if needed, is complete. |
| | Grade 3 or 4 elevation in AST, ALT, or total bilirubin | Permanently discontinue treatment. |
| Immune-related nephritis and renal dysfunction | Grade 2 or 3 creatinine elevation | Withhold dose(s) until creatinine returns to baseline and management with corticosteroids is complete. |
| | Grade 4 creatinine elevation | Permanently discontinue treatment. |

| | | |
|---|---|---|
| Immune-related endocrinopathies | Symptomatic Grade 2 or 3 hypothyroidism, hyperthyroidism, hypophysitis,<br>Grade 2 adrenal insufficiency<br>Grade 3 diabetes | Withhold dose(s) until symptoms resolve and management with corticosteroids (if needed for symptoms of acute inflammation) is complete. Treatment should be continued in the presence of hormone replacement therapy as long as no symptoms are present. |
| | Grade 4 hypothyroidism<br>Grade 4 hypophysitis<br>Grade 3 or 4 adrenal insufficiency<br>Grade 4 diabetes | Permanently discontinue treatment. |
| Immune-related skin adverse reactions | Grade 3 rash | Withhold dose(s) until symptoms resolve and management with corticosteroids is complete. |
| | Grade 4 rash | Permanently discontinue treatment. |
| | Stevens-Johnson syndrome (SJS) or Toxic epidermal necrolysis (TEN) | Permanently discontinue treatment. |
| Other adverse reactions | Grade 3 (first occurrence) | Withhold dose(s) until symptoms resolve. |
| | Grade 3 myocarditis | Permanently discontinue treatment. |
| | Grade 4 or recurrent Grade 3; persistent Grade 2 or 3 despite treatment modification; inability to reduce corticosteroid dose to 10 mg prednisolone or equivalent per day | Permanently discontinue treatment. |

**Management of Specific Adverse Events**

| Diarrhoea Management | | |
|---|---|---|
| Grade 1<br>Diarrhoea <4 stools a day over baseline | Asymptomatic colitis<br>Continue nivolumab as per protocol<br>Symptomatic management | Close monitoring for worsening symptoms.<br>Educate patient to report worsening immediately.<br>If worsens treat as Grade 2 or 3-4. |
| Grade 2<br>Diarrhoea 4-6 stools per day over baseline<br>IV fluids required for <24 hours<br>Not interfering with ADL<br>Colitis<br>Abdominal pain, blood in stool | Delay nivolumab<br>Treat symptomatically | If resumes to grade 1 or less: resume nivolumab.<br>If persists for >5-7 days or recurs: Start IV methylprednisolone 0.5 mg - 1mg/kg per day or oral equivalent.<br>If symptoms improve to grade 1, taper steroids over at least one month, consider antibiotic for opportunistic infections and restart nivolumab.<br>If persists or worsens after 3-5 days of steroids, treat as grade 3-4. |
| Grade 3-4<br>Diarrhoea (G3)<br>Stools ≥ 7 times more per day than baseline, incontinence, IV fluids needed more than 24 hours, interfering with ADLs<br>Colitis<br>(G3) Severe abdominal pain, peritoneal signs, medical intervention indicated<br>(G4) life threatening, perforation | Discontinue nivolumab<br>Methylprednisolone 1-2 mg/kg daily IV or PO equivalent<br>Add prophylactic antibiotics for opportunistic infections<br>Consider lower endoscopy | If improves: Continue steroids until at least grade 1 then taper steroids over at least one month.<br>If persists after 3-5 days of steroids or recurs add infliximab 5 mg/kg if no contraindication.<br>Infliximab should not be used in cases of perforation or sepsis. |
| **Renal Adverse Event Management** | | |
| Grade 1<br>Creatinine >ULN and more than baseline but not ≤1.5 x baseline | Continue nivolumab as per protocol | Monitor creatinine weekly.<br>If creatinine returns to normal, return to monitoring as per protocol.<br>If worsens treat as Grade 2 to 3 or 4. |
| Grade 2-3<br>Creatinine >1.5 baseline but ≤6 x ULN | Delay nivolumab<br>Monitor creatinine every 2-3 days<br>Start methylprednisolone 0.5mg-1mg/kg IV or PO equivalent.<br>Consider renal biopsy and nephrology | If improves to grade 1, taper steroids over at least one month, consider antibiotic for opportunistic infections and restart or nivolumab. Continue monitoring creatinine as per protocol<br>If persists or worsens after 7 days, treat as grade 4. |

| | | |
|---|---|---|
| Grade 4<br>Creatinine >6 x ULN | Discontinue nivolumab.<br>Monitor creatinine daily.<br>Methylprednisolone 1 mg -2 mg/kg daily IV or PO equivalent.<br>Refer to nephrologist<br>Consider renal biopsy | If improves: Continue steroids until at least grade 1 then taper steroids over at least one month.<br>Add prophylactic antibiotics for opportunistic infections. |
| **Pulmonary Adverse Events** | | |
| Grade 1<br>Radiographic changes only | Continue nivolumab as per protocol.<br>Monitor symptoms every 3 days.<br>Consider pulmonary and infectious disease referrals | Re-image at least every 3 weeks.<br>If worsens treat as Grade 2 or 3-4. |
| Grade 2<br>Mild to moderate new symptoms | Delay nivolumab.<br>Pulmonary referral.<br>Infectious disease referral.<br>Consider hospitalization.<br>Start methylprednisolone 1mg/kg iv or oral equivalent.<br>Consider bronchoscopy and biopsy | Re-image every 1- 3 days.<br>When symptoms improve to near baseline, taper steroids over at least one month, consider antibiotic for opportunistic infections and restart nivolumab.<br>If not improving after 2 weeks or worsening, treat as grade 3-4. |
| Grade 3-4<br>Severe new symptoms<br>New/worsening hypoxia<br>Life threatening | Discontinue nivolumab.<br>Hospitalize.<br>Pulmonary referral.<br>Infectious disease referral.<br>Methylprednisolone 2 mg-4 mg/kg daily IV or PO equivalent.<br>Add prophylactic antibiotics for opportunistic infection.<br>Consider bronchoscopy and biopsy. | If improves to baseline, then taper steroids over at least 6 weeks.<br>If not improving after 48 hours or worsening, add additional immunosuppression. |
| **Endocrine adverse event management** | | |
| Asymptomatic thyroid stimulating hormone (TSH) elevation | Continue nivolumab as per protocol<br>If TSH <0.5 x lower limit of normal (LLN) or TSH >2 x ULN or out of range at 2 subsequent tests, include fT4<br>and consider endocrinology referral. | |
| Symptomatic endocrinopathy | Evaluate endocrine function.<br>Consider pituitary scan.<br>If symptomatic with abnormal lab test or pituitary scan:<br>Delay nivolumab.<br>Methylprednisolone 1mg-2mg/kg daily per day IV or oral equivalent.<br>Replace hormone appropriately. If no abnormal laboratory test/normal pituitary scan:<br>Continue nivolumab. | Repeat laboratory test in 1-3 weeks/MRI in 1 month.<br>If improves with or without hormone replacement, taper steroids over at least one month, consider antibiotic for opportunistic infections and restart nivolumab.<br>Patients with adrenal insufficiency may need to continue steroids with mineralocorticoid component. |
| Suspicion of adrenal crisis | Delay or discontinue nivolumab.<br>Rule out sepsis.<br>Administer IV fluids. | |

| | | |
|---|---|---|
| Severe dehydration, hypotension, shock, out of proportion to current illness. | Stress dose of IV steroids with mineralocorticoid activity<br>Refer to endocrinologist.<br>If adrenal crisis ruled out treat as for symptomatic endocrinopathy above. | |
| **Neurological adverse events** | | |
| Grade 1<br>Asymptomatic or mild symptoms, intervention not indicated. | Continue nivolumab as per protocol. | Close monitoring for worsening symptoms.<br>If worsens treat as Grade 2 or 3-4. |
| Grade 2<br>Moderate symptoms<br>Limiting instrumental ADL | Delay nivolumab. Treat as per local guidelines.<br>Consider methylprednisolone 0.5mg-1mg/kg per day IV or PO equivalent. | If resolved to baseline resume nivolumab.<br>If worsens, treat as grade 3-4. |
| Grade 3-4<br>Severe symptoms,<br>Limiting self-care, ADL<br>Life threatening | Discontinue nivolumab.<br>Neurology referral.<br>Treat as per local guidelines.<br>Methylprednisolone 1mg - 2mg/kg daily or PO equivalent.<br>Add prophylactic antibiotics for opportunistic infections. | If improves to grade 2<br>taper steroids over at least one month.<br>If atypical or worsens, consider IVIG or other immunosuppressants as per local guidelines. |
| **Management of skin adverse reactions** | | |
| Grade 1 – 2<br>Covering less than 30% body surface area | Continue nivolumab as per protocol.<br>Symptomatic therapy (e.g. topical steroids, antihistamines)<br><br>If persists >1-2 weeks or recurs:<br>Consider skin biopsy,<br>Delay nivolumab,<br>Consider methylprednisolone 0.5mg-1mg/kg per day IV or PO equivalent. | If improves:<br>Taper steroids over at least one month, consider prophylactic antibiotics for opportunistic infections and resume nivolumab.<br>If worsens:<br>Treat as grade 3-4. |
| Grade 3-4<br>Covering >30% Body surface area,<br>Life threatening consequences | Discontinue nivolumab.<br>Dermatology referral.<br>Consider skin biopsy.<br>Methylprednisolone 1mg - 2mg/kg daily IV or PO equivalent | If improves to grade 1:<br>taper steroids over at least one month, consider prophylactic antibiotics for opportunistic infections. |
| **Management of hepatic adverse reactions** | | |
| Grade 1<br>AST or ALT > ULN to 3.0 x ULN and/or T.bili > ULN to 1.5 x ULN | Continue nivolumab as per protocol. | Continue LFT monitoring per protocol.<br>If worsens<br>Treat as Grade 2 or 3-4 |
| Grade 2 | Delay nivolumab as per protocol | If returns to baseline: |

| | | |
|---|---|---|
| AST or ALT > 3.0 to ≤5 x ULN and/or T.bili > 1.5 to ≤ 3 x ULN | Increase frequency of monitoring to every 3 days | Resume routine monitoring, resume nivolumab therapy per protocol.<br><br>If elevations persist > 5-7 days or worsen:<br>0.5-1mg/kg methylprednisolone IV or PO equivalent and when LFT returns to grade 1 or baseline, taper steroids over at least 1 month, consider prophylactic antibiotics for opportunistic infections and resume nivolumab therapy per protocol. |
| Grade 3-4<br>AST or ALT > 5 x ULN or T.bili > 3 x ULN | • Discontinue nivolumab.<br>• Increase frequency of monitoring to every 1-2 days<br>• 1mg – 2 mg/kg/day methylprednisolone IV or PO equivalent.<br>• Add prophylactic antibiotics for opportunistic infections<br>• Referral to gastroenterologist. | If returns to grade 2:<br>Taper steroids over at least 1 month.<br><br>If it does not improve in >3-5days, worsens or rebounds:<br>Add mycophenolate mofetil 1g BID<br>If no response within an additional 3-5 days, consider other immunosuppressants per local guidelines. |
| **Management of myocarditis adverse events** | | |
| Grade 2<br>Symptoms with mild to moderate activity or exertion | Delay nivolumab; hospitalisation with cardiac monitoring<br>Urgent cardiology referral for evaluation and management:<br>• Troponin and BNP<br>• ECG ± continuous cardiac monitoring<br>• Echocardiogram<br>• Cardiac MRI<br>Prompt initiation of 2mg/kg/day methylprednisolone IV or PO equivalent. | • If worsens, intensify treatment according to grade.<br>• Upon recovery, taper steroids over at least 1 month with close monitoring of troponin and BNP as well as for new symptoms.<br>• Repeat cardiac MRI for post treatment assessment and cardiology follow up.<br>• Retreatment may be considered after recovery and completion of steroid taper. |
| Grade 3<br>Severe with symptoms at rest or with minimal activity or exertion: intervention indicated<br><br>Grade 4<br>Life threatening consequences: urgent intervention indicated (e.g. Continuous IV therapy or mechanical hemodynamic support) | • Permanently discontinue nivolumab<br>• Hospitalise to intensive cardiac monitoring<br>• Cardiac evaluation to include:<br>- Troponin and BNP monitoring<br>- ECG ± continuous cardiac monitoring<br>- Echocardiogram<br>- Cardiac MRI<br>- Myocardial biopsy if feasible<br>• Immediate initiation of 2mg/kg/day | • If no improvement, consider additional immunosuppression.<br>• Upon recovery, taper steroids over at least 1 month with close monitoring of troponin and BNP as well as for new symptoms.<br>• Repeat cardiac assessment MRI for post treatment assessments and cardiology follow-up. |

| | | |
|---|---|---|
| | methylprednisolone IV or 1g IV bolus/day<br>• Consider adding a second immunosuppressive agent<br>Additionally, for Grade 4:<br>• Hospitalise /transfer to institution with expertise in intensive cardiac monitoring<br>• Consider ATG as second agent given its immediate effect | |

**Management of Nivolumab Infusion Related Reactions**

| **Management of infusion reactions** | |
|---|---|
| **Infusion reactions** | |
| Grade 1<br>Mild reaction, infusion interruption not indicated, intervention not indicated | Remain at bedside.<br>Monitor subject until resolution of symptoms.<br>Future infusions should receive pre-treatment with antihistamine (e.g. IV chlorphenamine) and/or paracetamol 1000 mg PO at least 30 minutes before infusion. |
| Grade 2<br>Moderate reaction required therapy or infusion interruption but responds promptly to symptomatic treatment; prophylactic medications indicated for ≤ 24 hours | Stop the nivolumab infusion.<br>Begin IV sodium chloride 0.9%.<br>Treat the participant with chlorphenamine 10 mg IV and/or paracetamol 1000 mg.<br>Remain at the bedside and monitor subject until resolution of symptoms.<br>Corticosteroid and/or bronchodilator therapy may also be administered if required.<br>Restart the infusion at 50% of the original infusion rate when symptoms resolve; if no further symptoms occur after 30 minutes, the rate may be increased to 100% of the original infusion rate.<br>Monitor subject closely.<br>If symptoms recur, then no further nivolumab will be administered at that visit.<br>For future infusions of nivolumab, the following prophylactic premedications are recommended: IV chlorphenamine 10 mg (or equivalent) and/or paracetamol 1000 mg PO should be administered at least 30 minutes before nivolumab infusion.<br>If necessary, corticosteroids (up to 25 mg of hydrocortisone or equivalent) may be used. |
| Grade 3 or 4:<br>Grade 3: prolonged (i.e., not rapidly responsive to symptomatic medication and/or brief interruption of infusion); recurrence of symptoms following initial improvement; hospitalization indicated for other clinical | Begin IV sodium chloride 0.9%.<br>Recommend bronchodilators, adrenaline 0.2 to 1 mg of a 1:1000 solution for subcutaneous administration or 0.1 to 0.25 mg of a 1:10,000 solution injected slowly for IV administration, and/or chlorphenamine 10 mg IV with methylprednisolone 100 mg IV (or equivalent), as needed.<br>Participant should be monitored until the Investigator is comfortable that the symptoms will not recur.<br>Nivolumab will be permanently discontinued. |

| | |
|---|---|
| sequelae (e.g., renal impairment, pulmonary infiltrates).<br>Grade 4: Life-threatening; pressor or ventilator support indicated | Investigators should follow their institutional guidelines for the treatment of anaphylaxis. Remain at bedside and monitor subject until recovery of the symptoms. |
