# Supplementary material for: ELEVATE – evaluating Temozolomide and Nivolumab in patients with advanced unresectable previously treated oesophagogastric adenocarcinoma with MGMT methylation: study protocol for a single arm phase II trial
Source: BMC Cancer. 2022 Sep 1;22:946. doi: 10.1186/s12885-022-09891-9 (PMC9434527; doi:10.1186/s12885-022-09891-9)
Supplement: Supplementary file 2 — Additional file 2 (Informed Consent Form for Tumour Analysis) PDF Format. Copy of the consent form given to participants for tumour analysis. [file 12885_2022_9891_MOESM2_ESM.pdf]

<TO BE PRINTED ON LOCAL HOSPITAL HEADED PAPER>

ELEVATE

INFORMED CONSENT FORM – TUMOUR SAMPLE SCREENING

Study Title: An open label phase II trial of temozolomide prior to nivolumab in MGMT deficient, advanced oesophagogastric cancer

Researcher: Dr Elizabeth Smyth

ERGO Ref: 61191

REC: 21/EE/0017

IRAS: 282284

Please initial each box

**1.**	I confirm that I have read and understand the Tumour Sample Screening patient information sheet dated [Insert here] for the transfer of my tumour sample to the Laboratories of Cambridge University. I have had the opportunity to ask questions and these have been answered satisfactorily.	☐
**2.**	I understand that my participation is voluntary and that I am free to withdraw at any time, without giving a reason and without my medical care or legal rights being affected.	☐
**3.**	I understand that relevant sections of my medical records may be looked at by the individuals from the Southampton Clinical Trials Unit (SCTU), the Sponsor or their delegates, from Regulatory Authorities, or from the NHS Trust where it is relevant to the transfer and storage of my tumour block. I give permission for these individuals to have access to my records.	☐
**4.**	I give my permission for a sample of my tumour, previously obtained for diagnostic purposes, to be sent to the laboratories of Cambridge University in order to determine where a MGMT deficiency is present.	☐

5. I understand that agreeing to the transfer and storage of my tumour block does not commit me to taking part in the ELEVATE trial. ☐

6. I understand that all information about me and the results will be held in strict confidence. ☐

7. I consent to the Southampton Clinical Trials Unit being informed of the result of my tumour sample analysis. ☐

8. I agree that the tumour sample and consent form will be stored on behalf of the ELEVATE Trial Management Group. If I choose to participate in the ELEVATE study, my tumour sample may be used in future ethically approved projects. I understand that some of these projects may be carried out by researchers other than the ELEVATE Trial Management Group. If I choose not to participate in the ELEVATE study, my tumour sample will be returned to my treating hospital and no further analysis conducted. ☐

9. I give permission for a copy of this consent form to be sent to the Southampton Clinical Trials Unit (where it will be kept securely), to allow confirmation of my consent. ☐

------------------------------------	------------------------------------	------------------------------
Name of Patient	Signature	Date

------------------------------------	------------------------------------	------------------------------
Name of Person taking consent	Signature	Date

REMINDER FOR RESEARCH TEAM:

- **Original signed consent form in Investigator Site File**
- **One copy given to the patient**
- **One copy filed in the patient's medical records**
- **One copy to be emailed to SCTU via secure nhs.net email account, safesend or encrypted mail to allow for central monitoring**
