# Supplementary material for: ELEVATE – evaluating Temozolomide and Nivolumab in patients with advanced unresectable previously treated oesophagogastric adenocarcinoma with MGMT methylation: study protocol for a single arm phase II trial
Source: BMC Cancer. 2022 Sep 1;22:946. doi: 10.1186/s12885-022-09891-9 (PMC9434527; doi:10.1186/s12885-022-09891-9)
Supplement: Supplementary file 3 — Additional file 3 (Informed Consent Form for the Main T rial) PDF Format. Copy of the consent form given to participants for the main trial. [file 12885_2022_9891_MOESM3_ESM.pdf]

ELEVATE

Informed Consent Form

An open label phase II trial of temozolomide prior to nivolumab in MGMT methylated, advanced oesophagogastric cancer

Researcher: Dr Elizabeth Smyth ERGO Ref: 61191 IRAS: 282284

Patient ID Number: | | | | | | | | | | | | |

Name of Researcher:

Please initial each box

1. I confirm that I have read and understand the patient information sheet dated **[date], [version]** for the above study and I fully understand what is involved in taking part in this trial. I have had the opportunity to ask questions and these have been answered satisfactorily. ☐

2. I understand that my participation is voluntary and that I am free to withdraw at any time, without giving a reason and without my medical care or legal rights being affected. ☐

3. I consent to the storage of personal information (including electronic) for the purposes of this study. I understand that any information that could identify me will be kept strictly confidential and that no personal information will be included in the study report or other publication. ☐

4. I understand that the information collected about me will be used to support other research in the future and may be shared anonymously with other researchers. ☐

5. I agree for my details to be registered with the National Health Service Digital or equivalent for which my name and NHS number must be used in order for my health status to be followed up.

6. I consent to give research blood and tissue sample(s) for use in laboratory research studies including genetic analysis: Blood DNA (extracted from blood). I understand that these samples will be sent to the laboratories at Cambridge University for analysis.

7. I agree that the blood samples, tissue samples, consent form and information collected about me will be stored on behalf of the ELEVATE Trial Management Group and may be used in future ethically approved research projects, which may include genetic testing. I understand that some of these projects may be carried out by researchers other than the ELEVATE Trial Management Group.

8. I agree to my anonymised data being used in future ethically approved research.

9. I agree to my GP being informed of my participation in the study.

10. I agree to my pseudo-anonymised data being held on servers located in the EU and USA. Access to data managed by Southampton Clinical Trials Unit (SCTU) will be strictly controlled and applicable Data Protection Legislation will be abided by.

11. I give permission for a copy of this consent form to be sent to the Southampton Clinical Trials Unit (where it will be kept securely), to allow confirmation of my consent.

12. I agree to use effective contraception as detailed in the patient information sheet and to refrain from donation of egg/sperm (if applicable) during the trial treatment and for 5 months (if female) or 7 months (if male) after the last dose of trial drug.

Yes **N/A**

13. I understand that I shall not benefit financially in any way by taking part in this study.

14. I understand that relevant sections of my medical records, and data collected during the study, may be looked at by individuals from the Sponsor or their delegates, from regulatory authorities, from the company supplying the drug or from the NHS Trust where it is relevant to my taking part in this research. I give permission for these individuals to have access to my records.

15. I agree to take part in the above study.

Name of Patient	Signature	Date
Name of Person taking consent	Signature	Date

REMINDER FOR RESEARCH TEAM:

- **Original signed consent form in Investigator Site File**
- **One copy given to the patient**
- **One copy filed in the patient's medical records**
- **One copy to be emailed to SCTU via secure nhs.net email account, safesend or encrypted mail to allow for central monitoring.**
